# Molecular dynamics simulation (MDS) analysis of *Vibrio cholerae* ToxT virulence factor complexed with docked potential inhibitors

**DOI:** 10.6026/97320630014101

**Published:** 2018-03-31

**Authors:** Ibrahim Torktaz, Ali Najafi, Reza Golmohamadi, Sorour Hassani

**Affiliations:** 1 Molecular Biology Research Center, Systems Biology and Poisonings institute, Baqiyatallah University of Medical Sciences, Tehran, IRAN

**Keywords:** ToxT, *Vibrio cholerae*, QSAR, g-mmpbsa, molecular dynamics simulation

## Abstract

The ToxT transcription factor mediates the transcription of the two major virulence factors in Vibrio cholerae. It has a DNA binding domain made of α4, α5, α6, α7, α8, α9 and α10 helices that is responsible for the transcription of virulence genes. Therefore, it is of interest to screen ToxT against the ZINC ligand database containing data for a million compounds. The QSAR model identified 40 top hits for ToxT. Two target protein complexes with ligands Lig N1 and Lig N2 with high score were selected for molecular dynamics simulation. Simulation data shows that ligands are stable in the DNA binding domain of ToxT. Moreover, Lig N1 and Lig N2 passed pharmacological as well as ADME filters along with g-mmpbsa analysis with binding affinity of -199.831 kJ/mol for Lig N1 and - 286.951 kJ/mol for Lig N2. Moreover, no Lipinski and PhysChem violations were identified. It is further observed that these compounds were not inhibitors of P-glycoprotein, CYP450 and renal organic cation transporters. The LD50 of 2.5804 mol/kg for Lig N1 and 2.7788 mol/kg for Lig N2 was noted with acceptable toxicity index.

## Background

*V. cholerae* is a gram-negative and facultative anaerobic pathogen that principally exists in aquatic locales. This is the causal agent of severe diarrhea by colonizing on the small intestine and secreting cholera toxin (CT). CT is a ribosylating toxin, which is responsible for the abundant diarrhea associated with cholerae. Secretion of CT by V. cholerae leads to a rise in cAMP levels in the host cells [1]. The elevation in cAMP concentrations results in the reduction adsorption of sodium, secretion of chloride ions into the lumen for naturalism and promotion of osmotic pressure, leading to voluminous secretion of water and electrolytes [2]. Toxin production occurs upon attachment of the bacteria onto the intestinal epithelium. Therefore, it is of interest to understand the regulatory mechanism of CT expression. Epidemic serotypes of V. cholerae are classical and El Tor where CT transcription is regulated by ToxT [3]. ToxT activates the transcription of ctxAB operon that encodes two CT subunits and the transcription of TCP operon encoding toxin-coregulated pilus genes [4]. Moreover, ToxT is an AraC family member, which includes the binding domain into the DNA with helix-turn-helix motifs and activates the expression of a number of virulence genes including TCP and CT [5]. Transcription of ToxT is activated with four inner-membrane proteins namely toxR, toxS, tcpP, and tcpH. Two chromosomally encoded regulators; AphB and AphA activate the transcription of tcpPH. Yamasaki et al. have shown that red chili and one of its active compounds capsaicin inhibit CT production without affecting bacterial growth [6]. Hung et al. revealed that virstatin inhibited ToxT activity when ToxT was expressed under the control of a heterologous pBAD promoter [7]. Plecha et al. showed that unsaturated fatty acids inhibited the DNA binding of ToxT [8]. Two available commercial oral cholera vaccines, ShanChol and Dukoral are available. They provide an impound protection of >50% for at least two years in indigenous population. However, they are not currently licensed in the united states [9]. Many V. cholerae strains have become restraint to a range of antimicrobial agents including tetracycline and ampicillin [10]. Thus, there is an essential need to develop new drugs against cholera. Shakhnovich et al. reported that virstain affects the ToxT activity of the ctx promoter by inhibition of ToxT dimerization [11]. Minto et al. revealed that malonate has the potential to inhibit the expression of disease-causing genes in V. cholerae through ToxT inhibition [12]. Therefore, it is of interest to screen ToxT against the ZINC ligand database containing data for a million compounds.

## Methodology

### Ligand screening

The 3D crystallography structure of ToxT from V. Cholera (PDB ID: 3GBG) was selected as the protein target in virtual screening model [13]. Molegro Virtual Docker (MVD) v 6.0 was used to calculate dock score and evaluate conformers. The DNA binding domain of ToxT identified and then the spherical virtual screening coordinate was located to this region. Nearly, 40000 drugs-like ligands were derived from subset 3_p0.1 from standard in stock drug like category by ZINC database and were used for virtual screening.

ZINC is a free database containing various compounds for docking based screening [14]. Docking parameters were set as follow: grid resolution of 0.3 Å for all docking simulation, a maximum number of 1500 iterations and each of 10 independent runs were enforced on single populations of 50 individuals. An 18 Å radius was exactly set on the coordinates of X: 40, Y: 55 and Z: 36 to cover the entire DNA binding site including α4, α5, α6, α7, α8, α9 and α10. Non-polar hydrogen atoms were removed from the receptor structure and their partial charges were added to the corresponding carbon atoms. Moreover, flexible torsions of ligands were identified by MVD. MolDock based on guided differential evolution and PlANTS scores performed docking simulations. The best conformations were based on the lowest binding energy.

### Pharmacophore model prediction and QSAR modeling

We selected the top 40 successive hits for structural alignment in order to find structurally similar ligands. The structure of hits that retrieved from virtual screening was largely diverse and five ligands with most similar structures were selected for pharmacophore design. Mastero 10.2 from Schrodinger simulation package was used for developing pharmacophore models following QSAR. The extracted common pharmacophore model was used for screening a local database containing 1,000,000 lead like small molecules.

### MD simulations:

GROMACS MD package 4.6.5 [15] was used to simulate the ToxT-ligand(s) interactions in a dynamic environment. The PRODRG server that is based on the GROMOS force field was employed to generate topology files for ligands [16]. GROMOS96 force field with a modified version of the 53a6.ff force field was utilized throughout the simulation study. The complexes of ToxT-ligand(s) were merged in a cubic-shaped box with the minimum distance of one nm between the protein surface and the box walls, followed by solvation in a simple point charge water molecules. Periodic boundary conditions were assigned the system was neutralized for energy minimization employing steepest descent algorithm with a tolerance of 1000 kJ/mol/nm. Equilibrations with harmonic restraints on the coordinates of the complexes atoms were performed after convergence. Parinello- Rahman barostat and modified Berendsen thermostat were applied to keep the pressure and temperature constant at 1 bar and 300 K, respectively. Particle-Mesh Ewald method was used to calculate Long-range electrostatic interactions. The MD runs were carried out in the NPT ensemble (50 ns) for each system. The binding affinities were calculated by g_mmpbsa package [17].

## Result and discussion

### Molecular docking study

**Figure 1:**
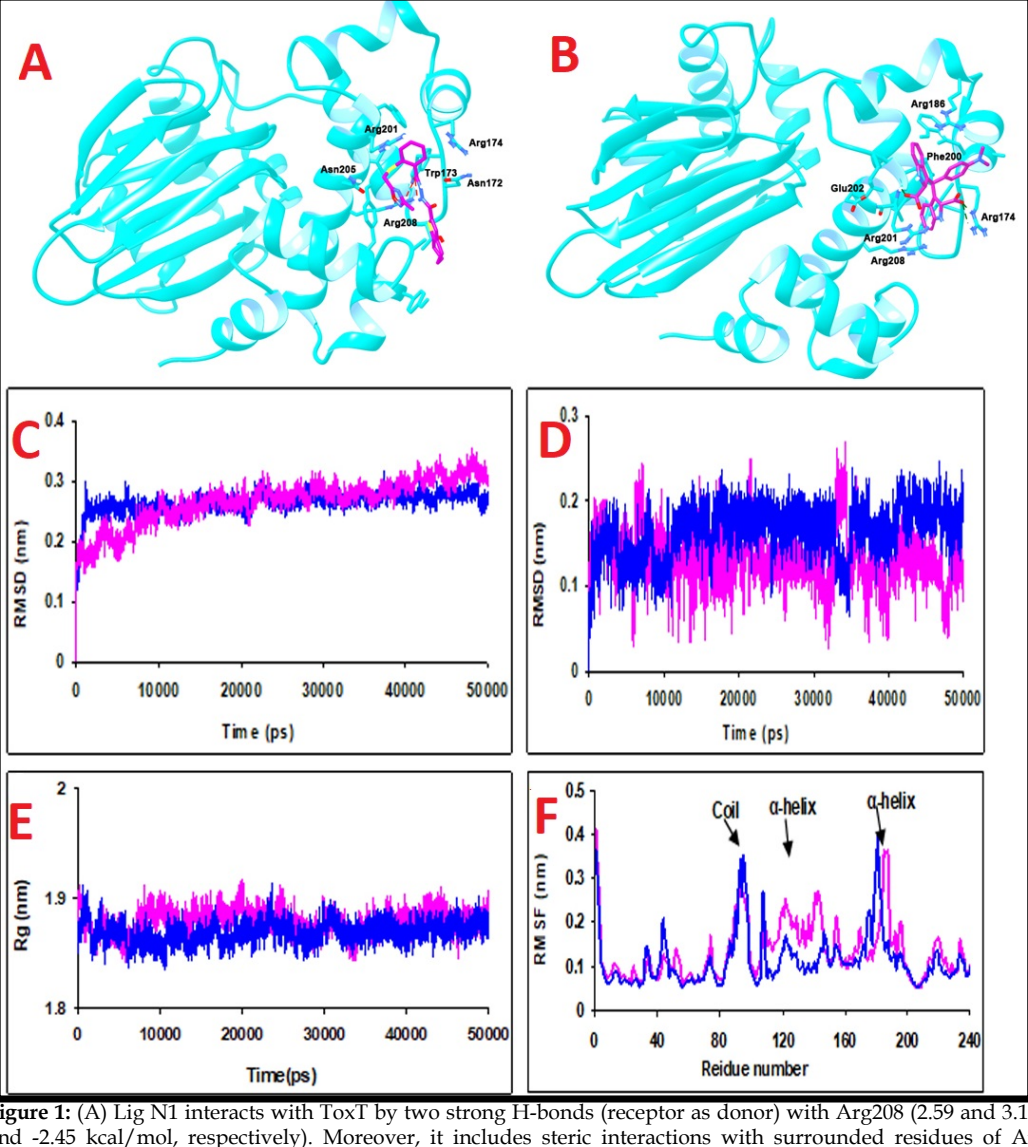
(A) Lig N1 interacts with ToxT by two strong H-bonds (receptor as donor) with Arg208 (2.59 and 3.12Å with energy of -2.5 and -2.45 kcal/mol, respectively). Moreover, it includes steric interactions with surrounded residues of Arg174, Trp173, Asn172, Asn205, Gly231, and Thr170 and π-π interaction with Phe200. (B) Lig N2 involved in hydrogen bindings with Arg201 and Arg174. one H-bonds with Arg201 (2.91 with energy of -2.07 kcal/mol) and two H-bond with Arg174 (2.9454Å and 2.54Å with energy -2.5 and - 0.89 kcal/mol, respectively). Moreover, Lig N2 include electrostatic interactions with Glu202 and Arg186, van der Waals interactions with Trp173, Phe200, Arg208, Ile204 and π-π interaction with Trp175. (C) RMSD value increases for ToxT -Lig N1 complex until; 0.28 nm, stayed around this value for; four ns, increases for a short while, and then stabilizes at; 0.32 nm. (D) RMSD value for ToxT-Lig N2 indicated that although the ligand is stable in the DNA Binding domain of ToxT, it has variation in RMSD, which means that it conformational alteration of the complex during MD simulation is much. (E) Rg of two protein-ligand complexes fluctuates around a stable value of 1.88 to 1.9 nm and does not show any significant drift. This low and nearly constant value of Rg approves good conformational stability and folding of two systems. (F) RMSF data indicated large fluctuations of segments belonging to the coils (95- 98 and 183-185) and α-helixes (90-94 and 185-189). While these features are common to both complexes, the ToxT structure exhibited a more fluctuations in segments of coil (125-127 and 146-146) and α-helix (128-145) when it binds to Lig N1. The RMSF values for residues (43-46), (94-97), and (173-183) are significant in ToxT-Lig N2 complex.

**Figure 2:**
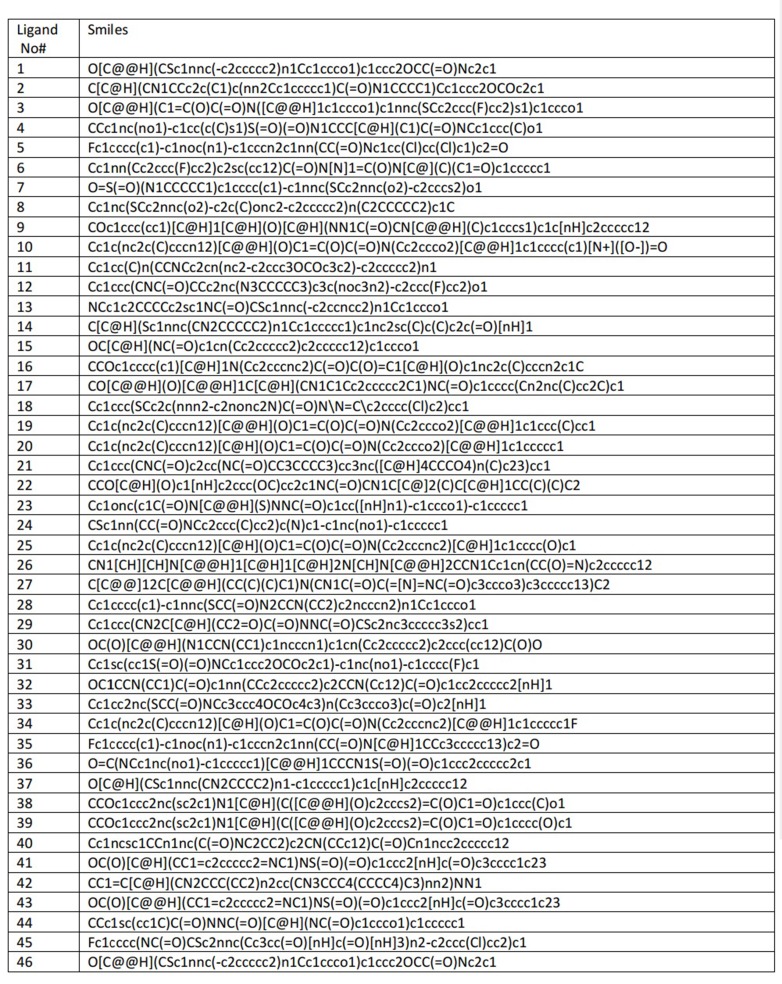
The results of top successive hits retrieved from virtual screening among 40.000 drug like small molecules from 3_p0.1 subset by a drug like category of Zinc database. MolDock and PLANTS are computational scores indicating binding affinity and are not relevant to chemical units.

The top 46 successive hits with the most efficient binding affinity are shown in Figure 2. Ligands that indicated Lipinski violation were excluded from the further study. The binding affinity of top 46 ligands was mostly in the same range but the structures indicated large variations. We used lig No# 1, 2, 3, 5 and 41 for a common pharmacophore model and QSAR prediction taking into consideration molecular similarity. The model was used for screening the ZINC database containing 1,000,000 lead like small molecules and the top hits were retrieved for a simulated docking study in an environment containing water molecules and neutralizing ions. Ligands, Lig N1 and Lig N2 showed the lowest binding energy with the receptor. The second docking screen with QSAR matched hits for new inhibitors of the regulatory protein of pathogenic operons in V. cholerae for these two ligands. Figure 1A shows Lig N1 interacts with ToxT by two strong Hbonds (receptor as a donor) with Arg208 (2.59 and 3.12Å with the energy of -2.5 and -2.45 kcal/mol, respectively). Moreover, it includes steric interactions with surrounded residues of Arg174, Trp173, Asn172, Asn205, Gly231, and Thr170 and π-π interaction with Phe200.

The residues of Arg201 and Arg174 are involved in hydrogen bonds with Lig N2. One H-bond with Arg201 (2.91 with the energy of -2.07 kcal/mol) and two H-bonds with Arg174 (2.9454Å and 2.54Å with energy -2.5 and -0.89 kcal/mol, respectively) are observed. Lig N2 includes electrostatic interactions with Glu202 and Arg186, van der Waals interactions with Trp173, Phe200, Arg208, Ile204 and π-π interaction with Trp175 (Figure 1B). Arg residue was the important residue involved in hydrogen bonding of receptor with ligands. The resulted data with scoring results showed that Lig N2 has more affinity to the receptor. Moreover, the RMSD of LigN1-receptor and LigN2-receptor is depicted in 1C and 1D, respectively. It showed that Lig N1 is more stable than Lig N2 in the DNA binding domain of ToxT. In addition, The Rg of both ligands (Figure 1E) indicated no significant drift. RMSF data (Figure 1F) indicated large fluctuations of segments in the coil regions (95-98 and 183-185) and α-helixes (90-94 and 185-189).

### Result of Drug-likeness and toxicity evaluation of the ligands

We checked the drug-likeness value of the top two virtual screening hits by FAF drugs [18] and admetSAR [19]. The results from FAFA-Drug and MolSoft indicated that Lig N1 was fitted into Lipinski acceptable area. Moreover, the oral absorption estimation indicated that Lig N1 has acceptable hydrogen bond donor/acceptor, rotatable bonds, molecular weight and hydrophobicity. The ligand encountered RO5 of drug-likeness without infringement. Lig N2 showed no Lipinski violation. In addition, the oral absorption estimation indicated that hydrogen bond donor/acceptor, rotatable bonds, molecular weight, and hydrophobicity are in an acceptable range. The results of AdmetSAR revealed that Lig N1 is non-inhibitor and nonsubstrate of P-glycoprotein, CYP450 and renal organic cation transporter. It was observed that these hits are not carcinogen and not toxic in ADMES assay. The LD50 of rat acute toxicity value for Lig N1 was predicted equally to 2.5804 mol/kg, which means that it is not toxic. Lig N2 was predicted as non-inhibitor of P-glycoprotein and renal organic cation transporter but it has predicted to be the substrate of P-glycoprotein with the probability of 0.6381. Moreover, it was predicted to be noninhibitor of CYP450 and not any carcinogenicity was predicted for it. Interestingly, it was predicted to be toxic in AMES assay. The LD50 of rat acute toxicity value for Lig N2 was predicted as 2.7788 mol/kg that puts Lig N2 in non-toxic chemicals category. From these two stages of docking screens and QSAR screening, Lig N1 and Lig N2 have selected to be taken into MD simulations as the next and final step in this evaluation process.

### g-mmpbsa analysis

g-mmpbsa package was used to calculate the binding affinity of ligands. By calculating potential energy in the vacuum, van der Waals, electrostatic interactions and net non-bonded potential energy between the protein and ligands were calculated. An average binding energy equal to -199.831 +/- 37.261 kJ/mol was achieved for Lig N1 and -286.951 +/- 48.611 kJ/mol for Lig N2 respectively. The binding affinities indicate that by attaching lig N1 and lig N2 to the DNA binding domain of ToxT, with the most probability, the protein cannot attach to the target DNA sequence.

## Conclusion

The molecular docking analysis of Lig N1 & N2 with the DNA binding domain of ToxT is shown in this report. The ligands docked with ToxT complex were stable over 50 ns molecular dynamics simulation. It is also noted that no violation of Lipinski riles and PhyChem were observed in the simulated target-ligand complexes.

## Conflict of interest

There is no conflict of interest.

## References

[1] Ramamurthy T (2003). Microbes Infect..

[2] Zahid MS (2010). Appl Environ Microbiol..

[3] DiRita VJ (1996). Proc Natl Acad Sci USA..

[4] Hsiao A (2009). Infect Immun..

[5] Jang J (2011). Microbiology..

[6] Yamasaki S (2011). Indian J Med Res..

[7] Hung DT (2005). Science..

[8] Plecha SC, Withey JH. (2015). J Bacteriol..

[9] Ali M (2017). Lancet Infect Dis..

[10] Dubief B (2017). Fish Shellfish Immunol..

[11] Shakhnovich EA (2007). Mol Microbiol..

[12] Minato Y (2013). PLoS One..

[13] Lowden MJ (2010). Proc Natl Acad Sci USA..

[14] Irwin JJ, Shoichet BK. (2005). J Chem Inf Model..

[15] Hess B (2008). J Chem Theory Comput..

[16] Schuttelkopf AW, van Aalten DM. (2004). Acta Crystallogr D Biol Crystallogr..

[17] Kumari R (2014). J Chem Inf Model..

[18] Miteva MA (2006). Nucleic Acids Res..

[19] Cheng F (2012). J Chem Inf Model..

